# Headache and Status Epilepticus Reveal Paget’s Disease of the Bone

**DOI:** 10.7759/cureus.60588

**Published:** 2024-05-19

**Authors:** Dahab Ouhabi, Houyam Tibar, Ali Benomar, Mohamed Jiddane, Wafa Regragui

**Affiliations:** 1 Department of Neurology B and Neurogenetics, Specialties Hospital, Ibn Sina Teaching Hospital, Mohammed V University in Rabat, Rabat, MAR; 2 Department of Neuroradiology, Specialties Hospital, Ibn Sina Teaching Hospital, Mohammed V University in Rabat, Rabat, MAR

**Keywords:** osteoporosis circumscripta, epilepsy, cotton wool sign, elderly headache, paget’s disease

## Abstract

Paget's disease of the bone (PDB) is a benign osteodystrophy of the elderly characterized by excessive remodeling of bone tissue, mainly in the pelvis, femur, and skull. Its neurological manifestations are numerous and affect both the central and peripheral nervous systems. As headaches are often reported, epileptic seizures remain exceptional. We report the case of a 75-year-old female patient with a history of chronic worsening headache who was admitted to the emergency department for the first episode of a seizure. Brain imaging revealed heterogeneous bone thickening and circumscribed skull osteoporosis. Bone scintigraphy showed pagetoid lesions restricted to the skull and face. Alkaline phosphatases increased. The rest of the biological work-up and the cerebrospinal fluid study ruled out other metabolic causes or central nervous system infections. The patient was treated with bisphosphonates and anti-convulsive treatment. The evolution was satisfactory, with progressive improvement in headache and seizure control, even several months after discontinuation of anti-seizure medication. Our case report highlights the importance of exploring chronic headaches in the elderly, not only in search of lesions of the cerebral parenchyma but also of the structures containing them, in this case, the skull.

## Introduction

Paget’s disease of the bone (PDB) is a chronic, progressive metabolic disease characterized by a focal bone resorption and formation disorder. It can manifest in either a monostotic or polyostotic form, affecting the pelvis, leg, skull, or spine. The PDB has a slow course and most of the time stays asymptomatic, though it may cause pain, deformities, pathological fractures, and osteosarcomas [1]. PDB cases are rarely reported in Africa, and the disease is most often encountered in elderly men. Its diagnosis is based on imaging, coupled with bone scintigraphy, which is recommended in all patients to evaluate the extent of the disease, and biochemical markers of bone turnover, which show markedly elevated serum alkaline phosphatase in the absence of liver disease [1]. Bisphosphonates are the treatment of choice for metabolically active PDB, along with a multidisciplinary approach for pain, deformities, and fractures. Paget’s disease of the skull has a wide range of central nervous system symptoms.

Headache in Paget’s disease is not rare but not specific and can reflect a variety of mechanisms, ranging from isolated thickening of the cranial vault to major therapeutic emergencies such as epidural hematoma with intracranial hypertension, hydrocephalus, cerebral herniation, platybasia, hydrocephalus, or dementia [2]. Epilepsy is a rare complication of Paget's disease. Its incidence is unknown, and its pathophysiology remains unclear [3].

## Case presentation

**Figure 1:**
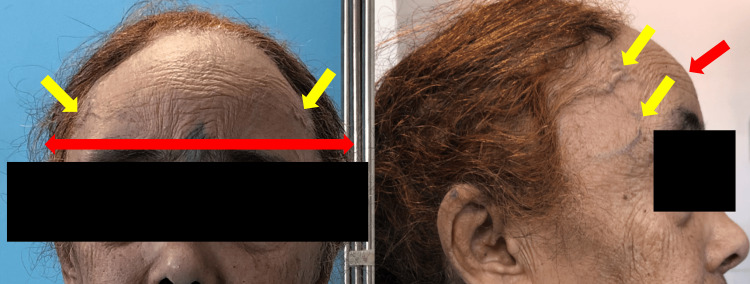
Frontal bossing features of the patient Wide and protruding asymmetric forehead (seen in red arrows) with temporal venous swelling (seen in yellow arrows).

We report the case of a 75-year-old female with a history of chronic diffuse, permanent, and isolated worsening headache, admitted to the emergency department for seizures. There was no history of neurovascular disease, neoplasm, seizures, head injury, cerebral infection, high blood pressure, or diabetes mellitus. Seizures began three weeks before admission, at a rate of one every other day, and were of the generalized tonic-clonic type, lasting about three minutes, without aura. No precipitating factors were identified. She was successfully treated with intravenous benzodiazepines. Her Glasgow Coma Scale (GCS) was 15/15. There was no fever or meningeal syndrome. Pupils were equal and reactive. We noted an enlargement of the temporal bones with increased head circumference, a protruding forehead, and bilateral temporal venous swelling (Figure 1).

**Figure 2:**
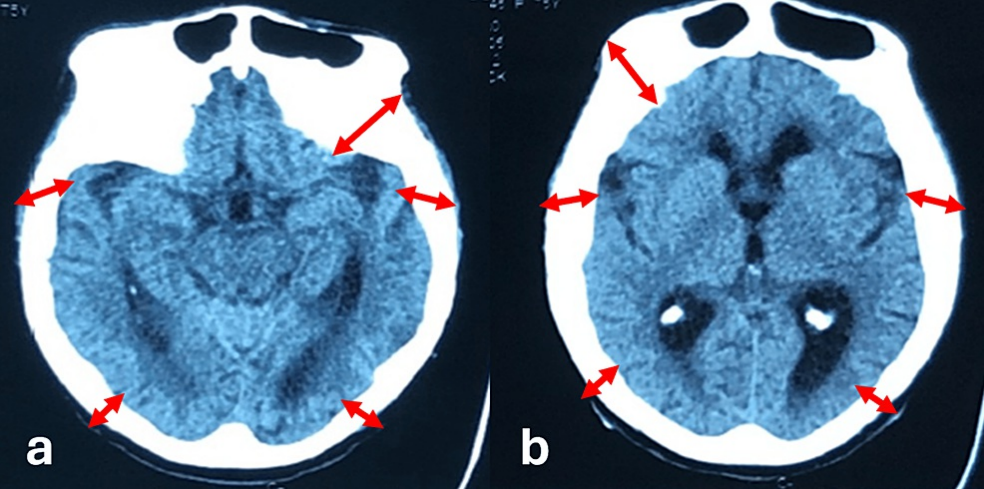
Brain axial CT scan without contrast Generalized thickening of the skull bones (red arrows).

Blood pressure and glucose levels were normal. The neurological examination was unremarkable. A brain CT revealed generalized skull bone thickening but no brain parenchyma abnormalities (Figure 2). Serum alkaline phosphatase levels were high (536 IU/L), with hyperparathyroidism (188 pg/mL) and blood calcium levels within the normal range. Metabolic workup was negative, including electrolyte panel, renal and liver function tests, and vitamin B9 and B12 levels. The inflammatory panel was negative. The cerebrospinal fluid (CSF) examination was normal. Polymerase chain reaction (PCR) panels in the CSF for infectious encephalitis and research for malignant cells were negative. An electroencephalogram (EEG) showed right frontal epileptic activity made of spikes and waves. Seizure control was obtained with levetiracetam (1000 mg/d).

On day five, the patient presented with fluctuating drowsiness. Another EEG showed signs of non-convulsive status epilepticus. She was treated with a bolus of levetiracetam.

**Figure 3:**
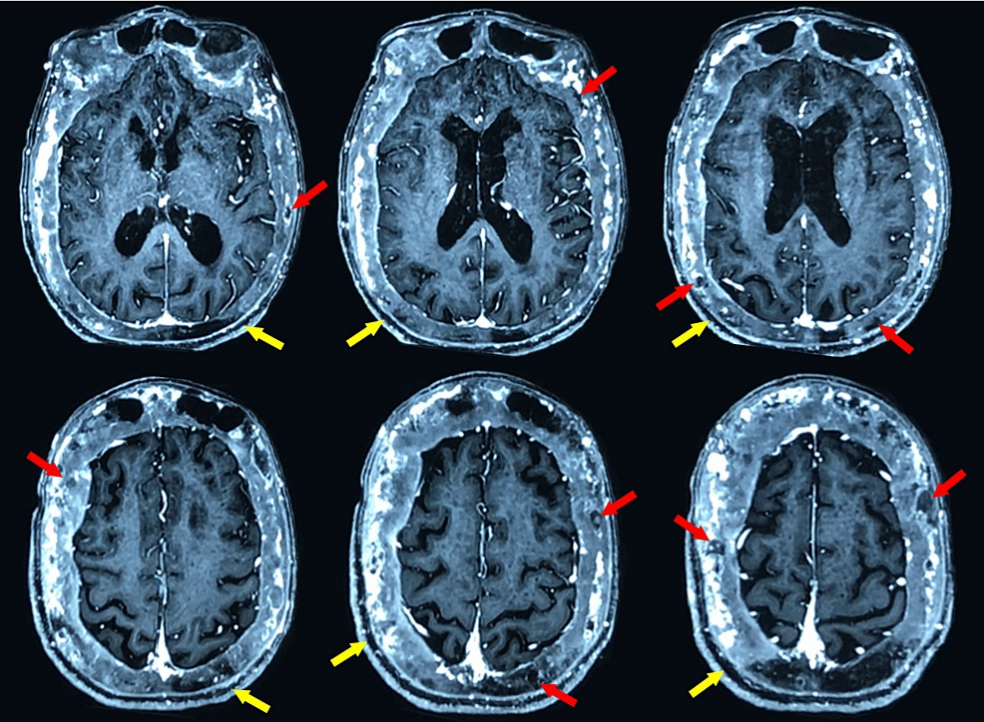
Axial T1-weighted post gadolinium brain MRI Osteoporosis circumscripta of the skull with large, lytic lesions (red arrows), a widened diploic space (yellow arrows), and right frontal lobe compression by bone thickening.

**Figure 4:**
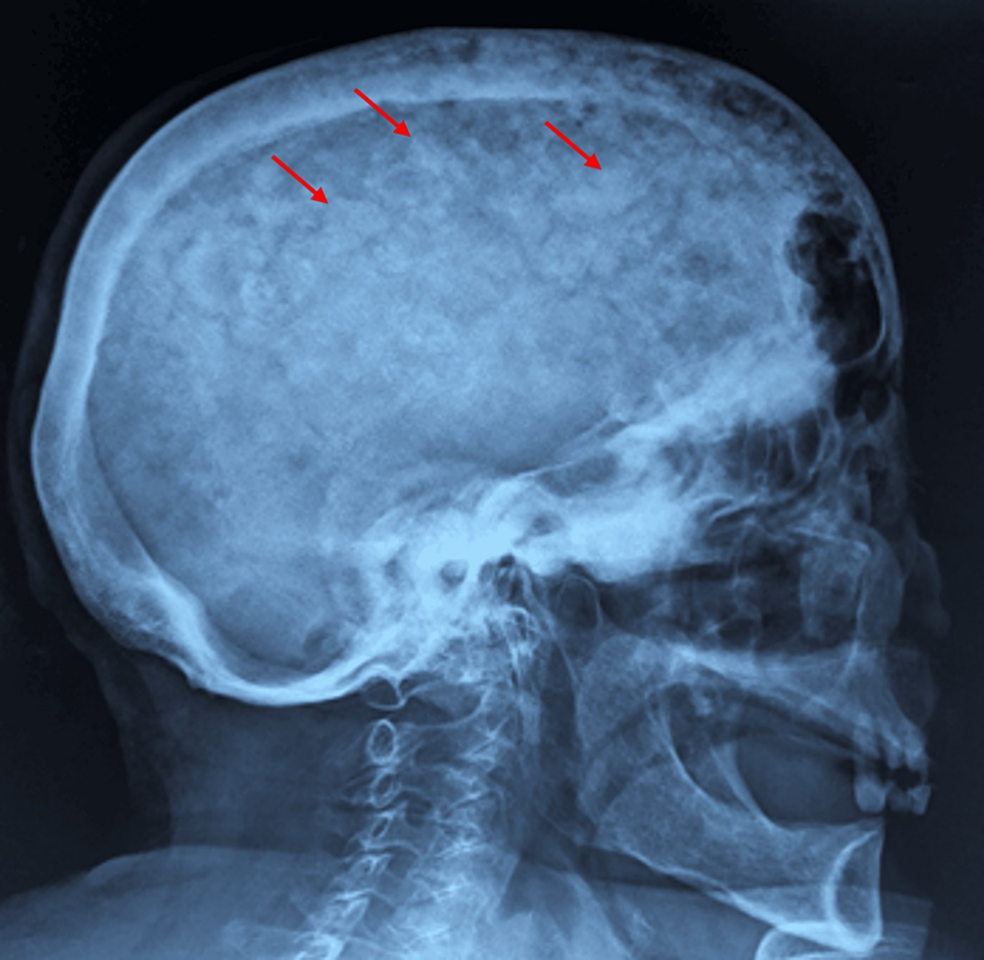
Skull X-ray Sclerotic patches (red arrows) give a cotton wool appearance to the skull.

**Figure 5:**
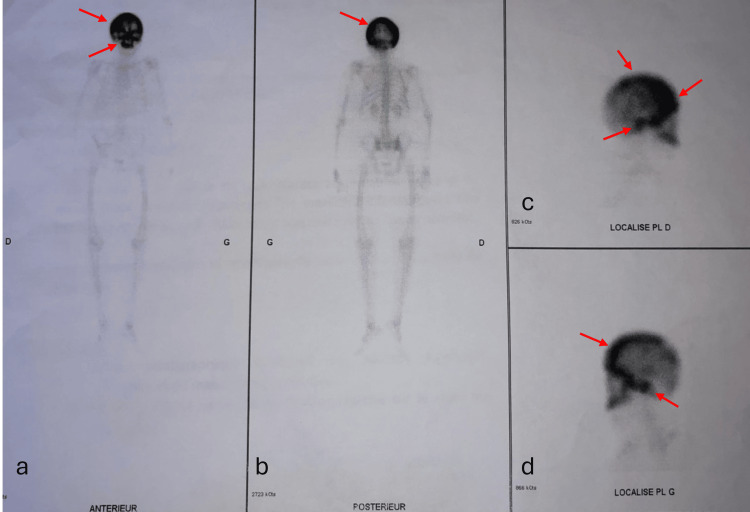
Total bone (a,b) and skull (c,d) scintigraphy Increased radionuclide uptake exclusively in the skull and facial massif (red arrows).

Brain MRI (Figure 3) showed diffuse osteoporosis circumscripta, large and lytic lesions, and diploic widening, especially near the right frontal lobe. The appearance and intensity of brain parenchyma were normal, but right frontal lobe compression by bone thickening was noticed. The ventricular system and cisternal spaces were normal. No intracranial space-occupying lesion or obvious vascular anomaly was detected. There was no shift in the midline structures. Skull X-ray showed a cotton wool appearance (Figure 4), and bone scintigraphy indicated increased radionuclide uptake in the skull and facial massif (Figure 5).

The patient received a single intravenous injection of zoledronic acid (5 mg) and kept levetiracetam (1000 mg/day) for three months before starting to taper the anti-seizure medication. The evolution was marked by the improvement of the headaches and complete seizure control since no epileptic seizures have occurred since the patient's non-convulsive status epilepticus.

## Discussion

PDB is a mono or polyostotic but never generalized bone disease with massive pathological remodeling of the bone, resulting in serious changes in shape, pain, fractures, and articular and neurological complications. The most affected areas are the pelvis, spine, skull, femur, and tibia. The disease is asymptomatic in 90% of patients in its early stages and is most often incidentally discovered [1]. The cause of PDB remains unclear, though some mechanisms were reported to have a certain role. PBD is strongly genetic, with 40% of patients having a history of similar cases in the family. More than 13 susceptibility genes have been associated with PDB. The most common mutation occurs in the SQSTM1 gene, which is located on chromosome 5q35 and codes for ubiquitin, a protein that plays a fundamental role in the growth and activation of osteoclasts. Environmental risk factors have also been identified in the etiopathogenesis of PDB, among which viral infections (measles, paramyxovirus, respiratory syncytial virus), and smoking [1].

Neurological complications occur in up to 76% of PDB cases and can happen at any stage of the disease. They can affect both the central and peripheral nervous systems [4]. The neurological signs of PDB manifest mainly in the fifth, sixth, and seventh decades of life, with a male predominance [5]. Ninety percent of the patients who present with neurological complications of PDB have more than one pagetic location [6]. Neurological presentation will occur depending on the location of the PDB. Deafness is the most common neurological complication of PDB, affecting up to 76% of patients, and is related to either auditory nerve compression, vascular shunting, or middle ear stiffening. Neck and back pain are also common, followed by spinal cord and radicular manifestations, which affect 2-5% of patients. The prevalence of cranial nerve damage varies widely, from 0.2% to 41%. Peripheral neuropathy is encountered in 2% to 5% of cases [5]. Headaches are considered to be uncommon and are found in 1.6% of cases [7], although they were reported in 67% of cases in one case series [8]. Other rare neurological complications exist, including epileptic seizures, as reported in some case studies [9-12].

Headaches in PDB are mostly posterior, intense, and aggravated by increased intracranial pressure (coughing, defecation). Several mechanisms may be involved. The main one is related to the hypervascularization of the skull bones, which is responsible for a regional vascular steal syndrome. Involvement of the skull base is responsible for platybasia and possibly basilar invagination leading to posterior headaches, hydrocephalus resulting in a headache, dizziness, and a gradually worsening dementia, eventually evolving towards a compression of the brainstem. Pagetoid compression of the trigeminal nerve can lead to trigeminal neuralgia. Thickening of the facial bones may lead to maxillary and/or mandibular bone deformities, leading to local pain and, by extension, headaches. Bone turnover of the skull can cause painful microfractures and may also increase the weight of the head and lead to occipital headaches because of the prolonged use of the cervical muscles to stabilize the position of the head [4]. Tumor degeneration of pagetoid lesions into osteosarcoma is a rare (< 1% of cases) but possible complication of PDB [13]. It presents with progressively worsening headaches associated with intracranial hypertension syndrome, exophthalmos, and focal signs depending on the topography of the tumor [5]. Intracranial hemorrhage is another cause of headaches in PDB. It may be spontaneous due to bone weakness or secondary to tumor bleeding [5].

Our patient developed neurological symptoms in her seventh decade. The chronic, isolated headaches were underestimated and did not prompt further investigation. It was the onset of epileptic seizures, a more severe neurological symptom, that led the patient to seek medical attention. Our hypothesis that epileptic seizures may have been the consequence of Paget's disease of the skull in our patient is based on the EEG abnormalities being localized to the right frontal region, which corresponds to the area most compressed by bone thickening on cerebral MRI; the occurrence of a non-convulsive status epilepticus while being on anti-seizure medication; the complete resolution of the seizures following the addition of zoledronic acid and the levetiracetam tapering; and the absence of a better explanation for the seizures, given the normality of the metabolic, infectious, inflammatory, and paraneoplastic work-up.

The occurrence of epileptic seizures in PDB is poorly reported in the literature [9-12], so their pathophysiology remains to be resolved. However, it can be assumed that mechanisms specific to the occurrence of headaches can be applied to epileptic seizures, such as regional vascular steal syndrome, tumor transformation, and intraparenchymal hemorrhage. In our patient's case, the right frontal diploid thickening led to adjacent cortical compression, with right frontal EEG epileptic abnormalities. Compression of the brain parenchyma near the bone turnover may be responsible for cortical suffering.

A few studies reported improvement in the neurological complications of PDB with bisphosphonates [4], which are the recommended treatment for all its locations. Zoledronic acid is the most effective [1]. Mild to moderate intermittent headaches are responsive to paracetamol and nonsteroidal anti-inflammatory drugs [14]. In the case of epileptic seizures, our patient responded well to levetiracetam, requiring it only for a few months while awaiting the administration of zoledronic acid before starting the anti-seizure medication tapering.

## Conclusions

There is a wide range of neurological complications in PDB. Seizures and headaches are rare manifestations and represent a challenge for the clinician because of their occurrence in the elderly, who often have associated affections that can manifest through similar clinical signs. However, some clues may point to the diagnosis of PDB, such as a positive family history of the disease, a physical examination showing a bulging of temporal veins or enlargement of the skull, progressive deafness, or a context of pain and bone deformities in other locations. The clinician should then perform a serum alkaline phosphatase assay as well as a brain MRI coupled with bone scintigraphy in the search for pagetoid lesions. The concern in diagnosing PDB is mainly the early initiation of bisphosphonates before debilitating or fatal complications occur.
